# Based on the Development and Verification of a Risk Stratification Nomogram: Predicting the Risk of Lung Cancer-Specific Mortality in Stage IIIA-N2 Unresectable Large Cell Lung Neuroendocrine Cancer Compared With Lung Squamous Cell Cancer and Lung Adenocarcinoma

**DOI:** 10.3389/fonc.2022.825598

**Published:** 2022-06-30

**Authors:** Ying Yang, Cheng Shen, Jingjing Shao, Yilang Wang, Gaoren Wang, Aiguo Shen

**Affiliations:** ^1^ Cancer Research Center Nantong, The Affiliated Tumor Hospital of Nantong University, Nantong University, Nantong, China; ^2^ Department of Computer Science and Engineering, Tandon School of Engineering, New York University, Brooklyn, NY, United States; ^3^ Department of Oncology, Nantong Tumor Hospital, Nantong University, Nantong, China; ^4^ Department of Radiology, Nantong Tumor Hospital, Nantong University, Nantong, China

**Keywords:** nomogram, lung cancer-specific survival, non-small cell lung cancer, histology, unresectable

## Abstract

**Background:**

The purpose of this study is to predict overall survival (OS) and lung cancer-specific survival (LCSS) in patients with stage IIIA-N2 unresectable lung squamous cell cancer (LUSC), lung adenocarcinoma (LUAD), and large cell neuroendocrine cancer (LCNEC) by constructing nomograms and to compare risk and prognostic factors affecting survival outcomes in different histological subtypes.

**Methods:**

We included 11,505 unresectable NSCLC patients at stage IIIA-N2 between 2010 and 2015 from the Surveillance, Epidemiology, and End Results (SEER) database. Moreover, competition models and nomograms were developed to predict prognostic factors for OS and LCSS.

**Results:**

Analysis of the SEER database identified 11,505 NSCLC patients, of whom 5,559 (48.3%) have LUAD, 5,842 (50.8%) have LUSC, and 104 (0.9%) have LCNEC. Overall, both OS and LCSS were significantly better in stage IIIA-N2 unresectable LUAD than in LCNEC, while there was no statistically significant difference between LUSC and LCNEC. Age, gender, T stage, chemotherapy, and radiotherapy were significantly associated with OS rates in LUAD and LUSC. However, chemotherapy was the only independent factor for LCNEC (*p* < 0.01).From competitive risk models, we found that older age, larger tumors, non-chemotherapy and non-radiotherapy were associated with a increased risk of death from LUAD and LUSC. Unlike prognostic factors for OS, our study showed that both chemotherapy and radiotherapy were all LCNEC-specific survival factors for both LCSS and non-LCSS LCNEC.

**Conclusion:**

Our study reports that unresectable patients with stage IIIA-N2 LCNEC and LUSC have worse LCSS than LUAD. The study’s first prognostic nomogram constructed for patients with unresectable stage IIIA-N2 NSCLC can accurately predict the survival of different histological types, which may provide a practical tool to help clinicians assess prognosis and stratify these prognostic risks to determine which patients should be given an optimized individual treatment strategy based on histology.

## Introduction

Lung cancer is the leading cause of cancer-related mortality worldwide. According to the latest data from the World Health Organization’s (WHO) International Agency for Research on Cancer (IARC), lung cancer has become one of the leading new cases and deaths worldwide in 2020. Non-small cell lung cancer (NSCLC) is the predominant histological type, accounting for approximately 85% of cases, with the majority of patients diagnosed at advanced unresectable stages (1). Of these, about 30% of NSCLC patients with IIIA or IIIB cannot be treated by surgical resection (2). The majority of patients who lose the chance of surgical treatment reportedly receive platinum-based chemotherapy (3). Patients with stage III non-surgical lung cancer achieve improved survival by modulating the dose of radiotherapy (4). These approaches provide partial remission, but patients’ 5-year overall survival (OS) remains suboptimal (5).

Chemotherapy or radiotherapy has been reported to exhibit tissue heterogeneity in the treatment of cancer patients. The most recent systematic evaluation analysis concluded that chemotherapy had different effects on OS in triple-negative breast cancer patients with different tissue subtypes (6). Jiang et al. demonstrated that the efficacy of chemotherapy was not statistically significant for signet ring cell cancer (SRCC) and adenocarcinoma (AD) in stage II colon cancer, whereas chemotherapy for stage III SRCC significantly reduced the risk of cancer-specific death (7). Another study found that radiotherapy for urothelial carcinoma improved OS in patients with AD and transitional cell carcinoma, but had no significant effect on squamous cell carcinoma (8). Based on these reports, histological subtype can play an important role in the selection of treatment and in predicting the survival outcomes of cancer patients.

NSCLC is known to include three subtypes: lung adenocarcinoma (LUAD), lung squamous carcinoma (LUSC), and large cell lung cancer (LCLC), with the former two being the most common ones. According to the 2015 Lung Tumor Classification by WHO, large cell neuroendocrine carcinoma (LCNEC) is a rare histologic lung cancer type in LCLC, with an incidence of approximately 3% (9). LCNEC has similar characteristics to small cell lung cancer with high invasiveness and recurrence rates and has a poor impact on patients’ survival (10). However, due to its low incidence, it has been rarely studied. The latest Clinical Guidelines for Non-Small Cell Lung Cancer (CSCO) state that LCNEC is a tissue subtype that differs from LUAD and LUSC. Therefore, it is necessary to explore the clinicopathological features, treatment, and prognosis of LCNEC.

Previous studies have found differences in tumor characteristics and prognosis among different tissue types of lung cancer (11). The efficacy of stereotactic body radiation therapy (SBRT) in patients with early-stage NSCLC showed histologically significant differences, and a multicenter study found that LUSC showed a worse OS status compared to LUAD (12). Previous studies have also found that patients with Phase III N2 LUSC treated with surgical resection after neoadjuvant chemotherapy with docetaxel-cisplatin (DP) had significantly better outcomes and survival rate than patients with LUAD (13). Not surprisingly, although clinical decision-making in NSCLC patients is still based on the tumor node metastasis (TNM) stage, the impact of different histologic subtypes on survival remains controversial. Recent literature reported the difference in postoperative survival of N2-III NSCLC patients with different histologic types and found that the OS rate of LUSC patients was worse than that of LUAD patients (14). However, whether different histological types affect the survival of unresectable stage IIIA-N2 patients is poorly defined.

Therefore, the main objective of this study is to assess how survival outcomes in inoperable stage N2-IIIA NSCLC patients vary by histologic subtypes. To assess independent risk factors for OS and lung cancer-specific survival (LCSS) for different histological subtypes, we developed a nomogram and competing risk model for unresectable patients with stage IIIA-N2 based on the SEER database.

## Method

### Study Design and Patient Selection

The NSCLC patients included in stage IIIA-N2 were cases from 2010 to 2015 (using the 7th edition of AJCC Cancer Staging Manual 7 classification). Corresponding details were taken from the SEER public access database and the SEER statistics version is 8.3.5.

Inclusion criteria were as follows: (a) NSCLC diagnosed as stage IIIA-N2 from 2010 to 2015; (b) histological subtypes only included LUAD, LUSC, and LCNEC; and (c) patients who could not undergo surgical resection excluding those who received surgical treatment.

Exclusion criteria were as follows: (a) patients with a follow-up period of less than 1 month or the follow-up time was not recorded in the SEER data; and (b) patients with incomplete clinicopathological or follow-up data.

### Statistical Analysis of Overall Survival

First, chi-square and *t*-tests were used to compare statistical differences in the proportions of the variables in the three groups with different tissue types. Survival curves drawn by the Kaplan–Meier method were used to compare the differences in the OS of the variables. Univariate and multivariate analyses were performed by Cox regression models, and independent risk factors were determined by multivariate analysis. Hazard ratios (HRs) and corresponding 95% confidence intervals (CIs) were calculated. Statistical analysis was performed by SPSS 26.0, and a *p-*value < 0.05 was considered statistically significant.

According to the significant independent risk factors, a nomogram model was established with survival and rms R-packages. The nomogram model was built and validated with guided internal verification. Discriminatory ability was determined by applying a Harmony Index (C-index). The 1-, 3-, and 5-year operating systems were calibrated to compare the predicted survival rate with the observed survival rate, and a calibration curve is provided. The above statistical analysis was performed using R version 4.1.0.

### Statistical Analysis of Lung Cancer-Specific Survival Rate

Cumulative incidence curves of lung cancer-related mortality (from the date of diagnosis) were constructed to compare LCSS with non-LCSS and to calculate mortality from other causes. Statistical comparisons of potential harms were performed using the Fine and Gray test (15). Using competing risk regression (Fine and Gray method), we analyzed risk factors for lung cancer-related mortality for three tissue subtypes, LUAD, LUSC, and LCNEC, including age, sex, T stage, histology, chemotherapy, and radiotherapy. We then used rms, cmprsk, and mstate R-packages to create corresponding nomograms for 1 year, 3 years, and 5 years of competitive risk models. *p*-values less than 0.05 were considered statistically significant.

## Results

### Clinical Characteristics of the Patient

**Figure 1:**
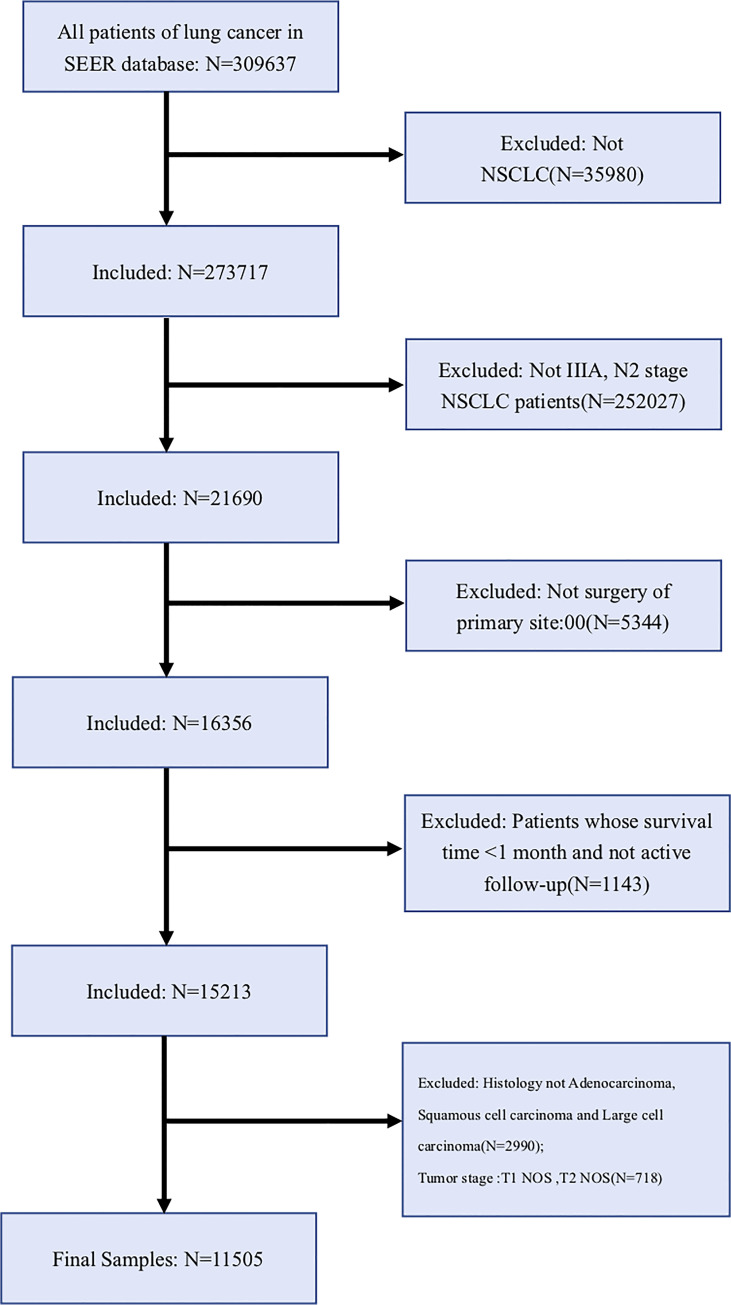
The flowchart for patient selection.

As shown in **Figure 1**, 21,690 NSCLC IIIA-N2 patients diagnosed between 2010 and 2015 were finally selected for this study, of which 11,505 met the inclusion criteria for the study. Based on the histological type of NSCLC, we divided unresectable IIIA-N2 patients into the LUAD group (5,559), the LUSC group (5,842), and the LCNEC group (104). In **Table S1**, we divided the included patients into three histological types and compared the basic demographic and clinicopathological characteristics of patients in the three cohorts.

### Independent Risk Factors for Overall Survival

**Tables 1**–**3** show univariate and multivariate analyses of OS in patients with unresectable IIIA-N2 in LUAD, LUSC, and LCNEC, respectively.

**Table 1:** Univariate and multivariate analyses for OS in patients with unresectable IIIA-N2 LUAD.

Characteristic Variables		Univariate		Multivariate	
		HR (95% CI)	*p*-value	HR (95% CI)	*p*-value
**Age**	20–39	Reference		Reference	
	40–59	0.506 (0.279, 0.918)	0.025	0.594 (0.327, 1.0790)	0.087
	60–79	0.566 (0.511, 0.626)	<0.001	0.693 (0.623, 0.770)	<0.001
	≥80	0.702 (0.65, 0.758)	<0.001	0.829 (0.765, 0.898)	<0.001
**Sex**	Female	Reference		Reference	
	Male	1.215 (1.142, 1.293)	<0.001	0.837 (0.786, 0.890)	<0.001
**Race**	White	Reference			
	Black	1.12 (0.989, 1.269)	0.075		
	Other	0.989 (0.854, 1.145)	0.882		
**Region**	East	Reference		Reference	
	Northern Plains	0.999 (0.934, 1.068)	0.968	1.078 (1.008, 1.153)	0.029
	Southern	0.9 (0.811, 1.000)	0.049	1.008 (0.908, 1.12)	0.878
	Alaska	1.353 (1.114, 1.642)	0.002	1.415 (1.164, 1.718)	<0.001
	Pacific Coast	3.69 (1.382, 9.850)	0.009	4.006 (1.500, 10.700)	0.006
**Grade**	I	0.833 (0.573, 1.209)	0.336		
	II	0.8 (0.537, 1.190)	0.271		
	III	0.85 (0.581, 1.242)	0.4		
	IV	0.973 (0.669, 1.415)	0.886		
	Unknown	Reference			
**Tumor Location**	Main bronchus	Reference			
	Upper lobe	0.904 (0.7, 1.168)	0.440		
	Middle lobe	0.817 (0.684, 0.975)	0.025		
	Lower lobe	0.803 (0.64, 1.007)	0.058		
	Overlapping	0.904 (0.754, 1.085)	0.279		
	NOS	0.858 (0.503, 1.464)	0.574		
**T stage**	T1a	Reference		Reference	
	T1b	0.641 (0.577, 0.713)	<0.001	0.617 (0.555, 0.686)	<0.001
	T2a	0.86 (0.781, 0.947)	0.002	0.790 (0.717, 0.870)	<0.001
	T2b	0.874 (0.806, 0.948)	0.001	0.831 (0.766, 0.902)	<0.001
	T3	0.991 (0.896, 1.096)	0.86	0.978 (0.884, 1.081)	0.661
**Chemotherapy**	No/Unknown	Reference		Reference	
	Yes	1.93 (1.808, 2.061)	<0.001	1.567 (1.454, 1.688)	<0.001
**Radiotherapy**	No/Unknown	Reference		Reference	
	Yes	1.75 (1.642, 1.866)	<0.001	1.466 (1.366, 1.573)	<0.001

**Table 2:** Univariate and multivariate analyses for OS in patients with unresectable IIIA-N2 LUSC.

Characteristic Variables		Univariate		Multivariate	
		HR (95% CI)	*p*-value	HR (95% CI)	*p*-value
**Age**	20–39	Reference		Reference	
	40–59	0.31 (0.116, 0.828)	0.019	0.36 (0.135, 0.962)	0.042
	60–79	0.618 (0.559, 0.683)	<0.001	0.787 (0.710, 0.872)	<0.001
	≥80	0.71 (0.662, 0.763)	<0.001	0.861 (0.800, 0.927)	<0.001
**Sex**	Female	Reference		Reference	
	Male	0.933 (0.879, 0.990)	0.022	0.915 (0.862, 0.972)	0.004
**Race**	White	Reference			
	Black	1.046 (0.913, 1.198)	0.517		
	Other	1.016 (0.87, 1.186)	0.842		
**Region**	East	Reference			
	Northern Plains	0.973 (0.914, 1.036)	0.396		
	Southern	0.896 (0.810, 0.990)	0.031		
	Alaska	1.106 (0.921, 1.328)	0.282		
	Pacific Coast	1.312 (0.760, 2.264)	0.330		
**Grade**	I	0.823 (0.559, 1.212)	0.325		
	II	0.819 (0.526, 1.276)	0.378		
	III	0.871 (0.59, 1.285)	0.486		
	IV	0.855 (0.58, 1.26)	0.428		
	Unknown	Reference			
**Tumor Location**	Main bronchus	Reference			
	Upper lobe	0.809 (0.653, 1.003)	0.053		
	Middle lobe	0.755 (0.63, 0.904)	0.002		
	Lower lobe	0.76 (0.601, 0.959)	0.021		
	Overlapping	0.915 (0.761, 1.101)	0.347		
	NOS	0.9 (0.63, 1.285)	0.562		
**T stage**	T1a	Reference		Reference	
	T1b	0.641 (0.564, 0.728)	<0.001	0.543 (0.477, 0.618)	<0.001
	T2a	0.772 (0.691, 0.863)	<0.001	0.720 (0.644, 0.806)	<0.001
	T2b	0.81 (0.755, 0.870)	<0.001	0.750 (0.698, 0.805)	<0.001
	T3	0.974 (0.989, 1.057)	0.526	0.943 (0.869, 1.024)	0.163
**Chemotherapy**	No/Unknown	Reference		Reference	
	Yes	2.028 (1.910, 2.152)	<0.001	1.714 (1.600, 1.836)	<0.001
**Radiotherapy**	No/Unknown	Reference		Reference	
	Yes	2.006 (1.886, 2.133)	<0.001	1.613 (1.507, 1.727)	<0.001

**Table 3:** Univariate and multivariate analyses for OS in patients with unresectable IIIA-N2 LCNEC.

Characteristic Variables		Univariate		Multivariate		
		HR (95% CI)	*p*-value	HR (95% CI)	*p*-value
**Age**	20–39	/			
	40–59	Reference			
	60–79	0.494 (0.247, 0.990)	0.047		
	≥80	0.629 (0.348,.1.139)	0.126		
**Sex**	Female	Reference			
	Male	0.717 (0.466, 1.103)	0.130		
**Race**	White	Reference			
	Black	0.384 (0.052, 2.813)	0.346		
	Other	0.4231 (0.056, 3.329)	0.420		
**Region**	East	Reference			
	Northern Plains	1.276 (0.810, 2.009)	0.293		
	Southern	0.796 (0.243, 2.613)	0.707		
	Alaska	/			
	Pacific Coast	1.220 (0.165, 9.006)	0.845		
**Grade**	I	0.738 (0.315, 1.730)	0.484		
	II	/			
	III	5.948 (0.682, 51.894)	0.107		
	IV	0.830 (0.341, 2.018)	0.681		
	Unknown	Reference			
**Tumor Location**	Main bronchus	Reference			
	Upper lobe	2.335 (0.575, 9.479)	0.236		
	Middle lobe	0.779 (0.281, 2.164)	0.632		
	Lower lobe	1.615 (0.450, 5.799)	0.463		
	Overlapping	0.859 (0.293, 2.523)	0.859		
	NOS	1.162 (0.129, 10.486)	0.894		
**T stage**	T1a	Reference			
	T1b	1.243 (0.663, 2.330)	0.499		
	T2a	1.188 (0.596, 2.369)	0.625		
	T2b	0.870 (0.489, 1.548)	0.636		
	T3	1.759 (0.838, 3.691)	0.135		
**Chemotherapy**	No/Unknown	Reference		Reference	
	Yes	3.276 (1.969, 5.450)	<0.001	3.276 (1.969, 5.450)	<0.001
**Radiotherapy**	No/Unknown	Reference			
	Yes	2.102 (1.339, 3.301)	0.001		

In the LUAD group, univariate analysis showed that age, gender, region (South, Alaska, and Pacific coast), tumor location (middle lobe), T stage (T1b, T2a, and T2b), chemotherapy, and radiotherapy were significantly associated with OS (*p* < 0.05). Multivariate analysis using Cox regression found that age, gender, region (Northern Plains, Alaska, and Pacific Coast), T stage (T1b, T2a, and T2b), chemotherapy, and radiotherapy were independent risk factors for LUAD (*p* < 0.05).

Univariate analysis in the LUSC group showed that age, gender, region (South), tumor location (middle and lower lobe), T stage (T1b, T2a, and T2b), chemotherapy, and radiotherapy were significantly different from OS (*p* < 0.05). Multivariate analysis using Cox regression showed that age, gender, T stage (T1b, T2a, and T2b), chemotherapy, and radiotherapy were independent risk factors for LUSC (*p* < 0.05).

In the LCNEC group, univariate analysis showed that age (60–79 years), chemotherapy, and radiotherapy were significantly different from OS (*p* < 0.05).The multivariate analysis with Cox regression showed that only chemotherapy was an independent risk factor for LCNEC (*p* < 0.05).

### Overall Survival Prognostic Analysis of Patients With Unresectable Stage

OS was better in LUAD than in LUSC and LCNEC, but there was no significant difference between LUSC and LCNEC (*p <*0.001) (**Figure S1**). According to our multivariate analysis, as shown in **Figure S2**, age (*p* < 0.05), sex (*p <*0.05), T stage (*p <*0.001), chemotherapy (*p <*0.001), and radiotherapy (*p <*0.001) were significant prognostic factors of OS in the LUAD and LUSC groups. Among patients with LUAD and LUSC, younger age, female, early T stage, and incorporating chemotherapy and radiotherapy had better OS. However age, sex, and T stage were not significantly related to LCNEC. In the LCNEC group, patients who received chemotherapy or radiotherapy had a better OS rate than those who did not receive treatment (*p <*0.001).

### Creation and Verification of Nomograms

**Figure 2:**
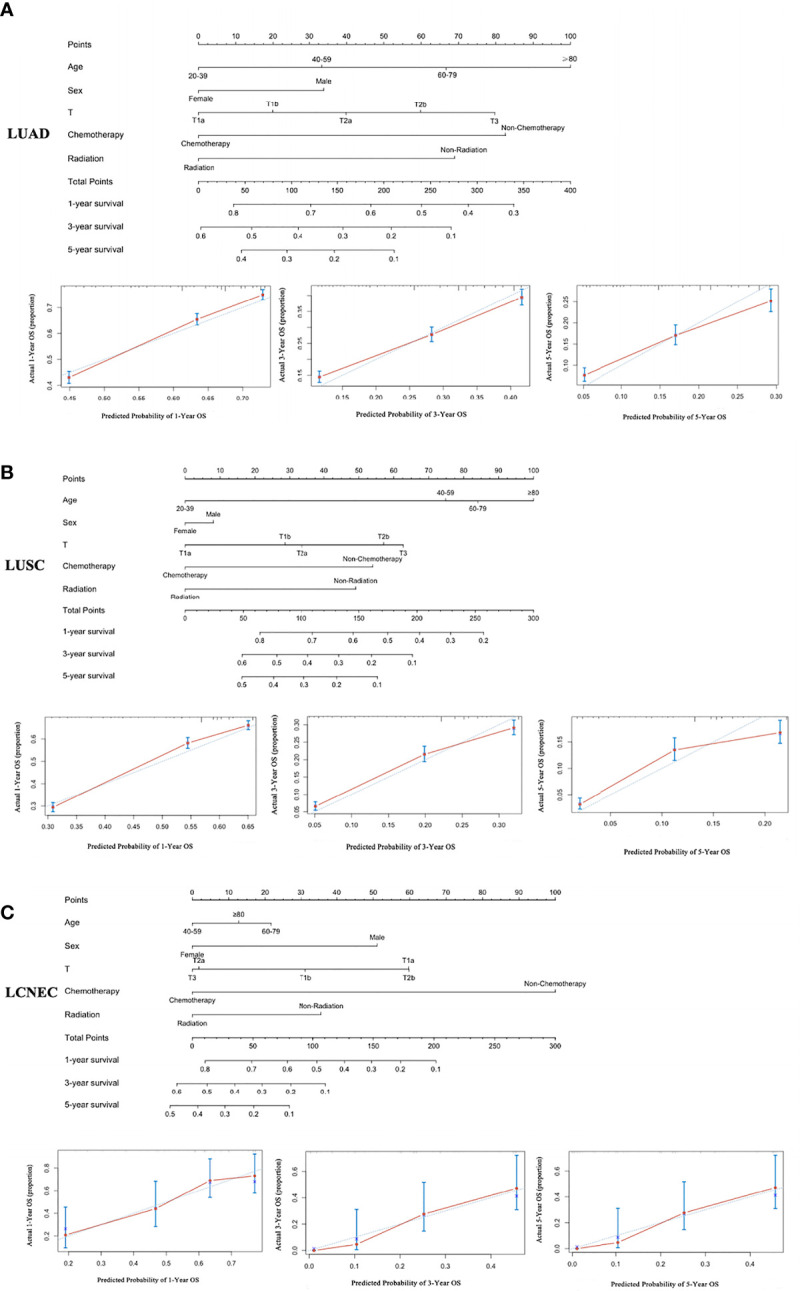
A nomogram for the prediction of 1-, 3-, and 5-year OS rates and the corresponding calibration curve: **(A)** LUAD, **(B)** LUSC, and **(C)** LCNEC.

Based on the independent risk factors obtained from Cox regression analysis, the nomogram was constructed to explore the OS rate of 1, 3, and 5 years for patients who were in an unresectable stage in IIIA-N2 (**Figure 2**). The calibration curves for the LUAD, LUSC, and LCNEC groups showed good agreement between the predictions of the nomograms and the actual observations of the OS at 1, 3, and 5 years. We examined the discrimination against nomograms, showing good predictive accuracy and clinical applicability, with C-index values of 0.638, 0.649, and 0.688 for the three groups, respectively.

### Prognostic Factor Analysis of LCSS

**Figure 3:**
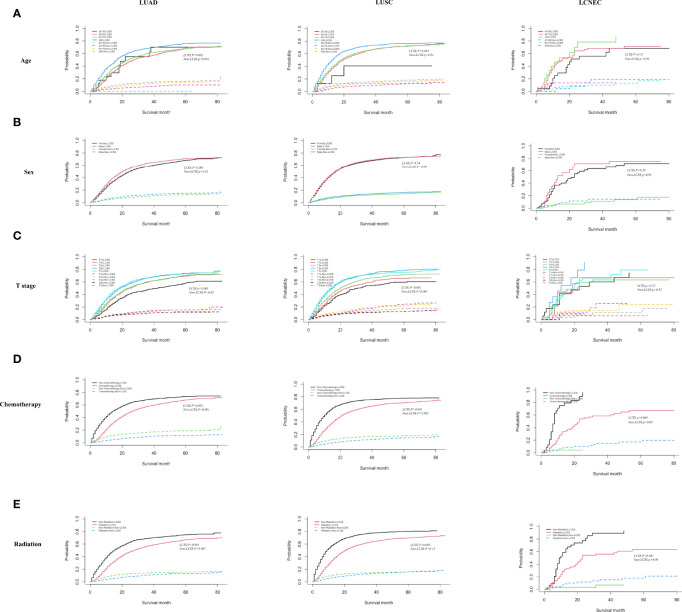
Competing risk analyses for patients in the LUAD, LUSC, and LCNEC group according to **(A)** age, **(B)** sex, **(C)** T stage, **(D)** chemotherapy, and **(E)** radiation.

In **Figure 3**, we proceeded to divide the patients into three different tissue subtypes to explore the cumulative risk of LCSS in each tissue subtype under different factor stratifications. As shown in **Figure 3A**, there was a statistically significant difference (*p* < 0.001) in the cumulative incidence of LCSS across age groups in LUAD and LUSC after controlling for competing risk events. In particular, the ≥80 years age group was significantly higher than the other groups (60–79, 40–59, and 20–39 years age groups). However, in the LCNEC group, there was no statistical significance (*p* = 0.32). As shown in **Figure 3B**, when patients were grouped by gender, the cumulative incidence of LCSS was significantly higher in male than in female patients in the LUAD group alone (*p* < 0.001), whereas there was no statistically significant difference between the cumulative incidence of LCSS in the LUSC and LCNEC groups (*p* = 0.53 and *p* = 0.28, respectively). Similarly, when stratified by T stage after controlling for competing risk events, the differences in the cumulative incidence of LCSS between the LUAD and LUSC groups were statistically significant for T1a, T1b, T2a, T2b, and T3, with patients in T1a having a lower cumulative incidence of LCSS than in T1b, T2a, T2b, or T3 (*p* < 0.001, **Figure 3C**). However, there was no difference in the LCNEC group (*p* = 0.52). In addition, the cumulative incidence of LCSS was significantly lower in patients receiving chemotherapy and radiotherapy than in those not receiving chemotherapy and radiotherapy in all three subtypes (*p* < 0.001, **Figures 3D, E**).

### Constructing a Prognostic Nomogram for the Competitive Risk Model

**Figure 4:**
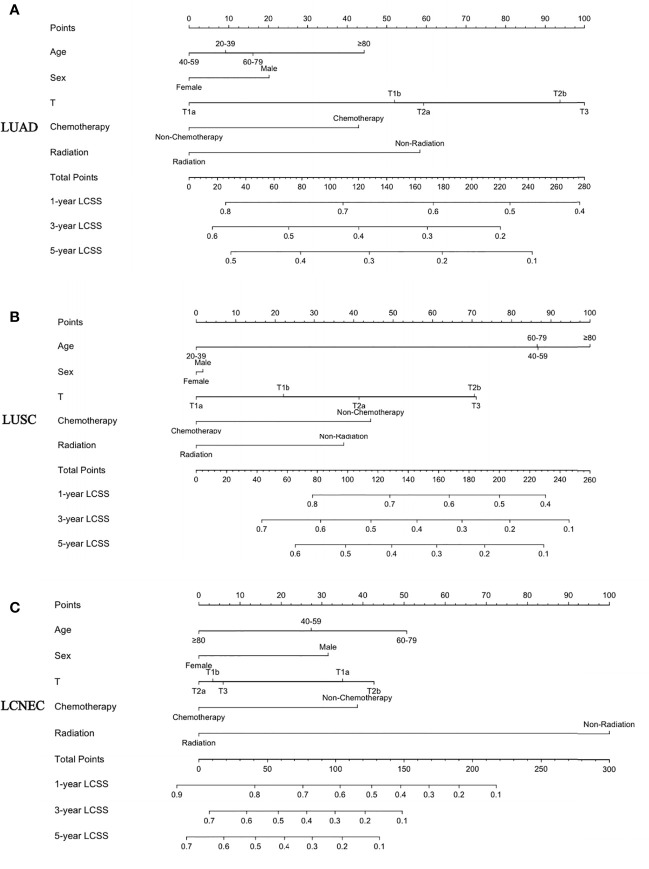
A nomogram for the prediction of 1-, 3-, and 5-year LCSS rates: **(A)** LUAD, **(B)** LUSC, and **(C)** LCNEC.

The nomogram for predicting LCSS is based on five independent risk factors: age, sex, T stage, chemotherapy, and radiotherapy (**Figure 4**). Each independent risk factor corresponds to a specific score by drawing a line on the dotted axis. The total score reflects the sum of the scores for each factor and is drawn directly down from the total point axis to the LCSS axis at 1, 3, and 5 years, corresponding to the predicted probability of LCSS at 1, 3, and 5 years.

## Discussion

In this study, we analyzed the survival of 11,505 patients with unresectable NSCLC (LUAD, LUSC, and LCNEC) diagnosed between 2010 and 2015 according to histologic subtypes in the SEER database. Based on statistical methods, we evaluated independent predictors of OS in patients with stage IIIA-N2 NSCLC of three histological subtypes. Three nomograms were constructed using the above factors to quantify survival at 1, 3, and 5 years. Calibration analyses were performed to assess the accuracy and validity of these line graphs. Finally, by controlling for competing risk events, we assessed the cumulative incidence of competing risks in the three tissue subtypes and constructed competing risk models and nomograms based on independent predictors. Although several models are available to predict the prognosis of lung cancer, a risk model focusing on different histological subtypes in unresectable IIIA-N2 stage patients has not been developed for patients with NSCLC. Therefore, the aim of this study was to develop a practical survival prediction model for individualized prediction of survival in patients with unresectable stage IIIA-N2 NSCLC.

Whether histologic subtype affects patient outcomes and survival is controversial. To reduce bias in this study, we included patients with LUSC, LUAD, and LCNEC as defined according to the AJCC 7th edition guidelines to ensure that most patients were treated in a relatively consistent and modern manner. In our study, age, gender, T stage, chemotherapy, and radiotherapy were all independent risk factors for LUAD and LUSC. Our results are consistent with those of recent years exploring the impact of tissue staging on survival and prognosis in NSCLC. The OS was significantly higher in LUAD than in LUSC, and LCSS was significantly lower in LUSC than in LUAD after controlling for competing risks (16, 17). Several studies have shown that LUSC is one of the most aggressive cancers, with a 5-year survival rate of only 10%. Smoking and alcohol consumption are important factors for the low survival rate and accelerated tumor progression in LUSC, which has been confirmed by many studies (18, 19).

To further determine the cause of the survival difference between LUAD and LUSC, a competing risk model was developed. The competing risk model was able to make better clinical predictions than the traditional Kaplan–Meier and Cox regression models. Age, gender, chemotherapy, and radiotherapy were independent risk factors for LCSS in the LUAD and LUSC groups. Compared with the older group, younger non-surgical NSCLC patients had significantly different LCSS, and after controlling for competing risk events, male LUAD patients had significantly lower LCSS than women, but there was no significant difference between the LUSC and LCNEC groups. Age is an important factor influencing lung cancer survival (20). Arnold et al. also found that the OS rate of NSCLC patients and the LCSS rate were significantly better in younger patients than in older patients (21). However, a 2015 retrospective study in Germany found that female lung cancer patients had significantly higher survival rates than men, but they stated that the high survival rates in women were independent of histology (22). This study found that female patients were more likely to develop AD, while male patients tend to suffer from squamous cell carcinoma. Perhaps due to the increased number of women smoking and the sensitivity of women to nicotine, there was no significant difference in the incidence of squamous cell carcinoma similar to the findings of Wheatley et al. (23). It is not difficult to find that age and gender are indeed important factors affecting LUAD and LUSC in this study, but due to the heterogeneity of the survey population, there are many factors that affect the survival of patients, which makes it worthy to provide more targeted and personalized treatment options for lung cancer patients in future clinical treatments and prospective studies.

Previous studies have found that LCNEC, a rare neuroendocrine carcinoma, has a lower survival rate than other NSCLCs (24, 25). In our study, a histological subtype of LCNEC was also available, and we found a significantly lower survival rate than LUAD from the survival curves, but there was no difference in the OS rate with LUSC. This may be due to the fact that only 104 patients with LCNEC met the inclusion criteria for this study, and it is difficult to develop a statistically significant trend due to the small sample size. Although there are fewer studies on unresectable LCNEC, a retrospective study found better survival in patients with stage IA or IB LCNEC who received chemotherapy after surgical resection (26). In an early case report, a combination of irinotecan and fractionated-dose cisplatin chemotherapy was found to be significantly more effective in older LCNEC patients (27). In this study, by controlling for competing risks, we also found that patients with stage IIIA-N2 unresectable LCNEC who received chemotherapy had a higher LCSS rate compared to those who did not receive chemotherapy.

Nomograms for OS and LCSS were constructed based on three tissue subtypes to provide more refined and personalized survival predictions for physicians and patients. In our study, chemotherapy and radiotherapy were independent risk factors associated with LCSS with important histologic subtypes. A retrospective study of the outcomes of unresectable stage III NSCLC patients treated with chemotherapy and radiotherapy from 2000 to 2013 also found that chemotherapy and radiotherapy were important in improving survival (5). In addition, with the overall development of lung cancer treatment, an increasing number of therapeutic approaches and predictors have shown great potential in improving patient survival and modeling lung cancer prognosis. The study by Antonia et al. found that the addition of immunotherapy to chemotherapy in patients with stage III unresectable NSCLC significantly improved OS (28). A recent study reported that gefitinib combined with pemetrexed and carboplatin chemotherapy significantly improved both treatment efficacy and survival in EGFR-mutant advanced NSCLC (29). Therefore, in addition to chemotherapy and radiotherapy, targeted therapy, immunotherapy and combination therapy may provide more personalized and specialized options to improve patient survival. Furthermore, a 2021 study by Avanzo et al. found that the application of radiomics is of great value in improving lung stereotactic body radiation therapy (30). In clinical practice, radiomics is an emerging field of research, and it is used as a predictive tool for responses and treatment outcomes. It may be a new strategy to predict the efficacy of radiotherapy in lung cancer patients, providing new ideas for patients to choose the best treatment regimen.

In addition, based on nomograms and competing risk models, we can more accurately distinguish and predict the survival of patients with different T stages. Our study found that after controlling for competing risk events, stage T1a patients had lower cumulative morbidity in the LUAD and LUSC groups than patients at stage T1b, T2a, T2b, or T3. Using the nomogram, we could also predict the 1-, 3-, and 5-year survival scores of patients with different T stages based on inoperable stage IIIA-N2 NSCLC patients.

However, our study has several limitations. Firstly, our study was retrospective and included Americans and heterogeneous individuals. Secondly, the treatment records in the SEER database did not contain information on the name of chemotherapy drugs, number of chemotherapy treatments, radiation dose, and number of radiation treatments. Thirdly, the database did not contain important clinical information, such as smoking history, lymphovascular invasion, neural invasion, cancer thrombosis, tumor recurrence, and related treatments. Fourthly, tumor mutation-driver genes such as EGFR, ALK, and ROS1 and the use of targeted therapies were not recorded in the SEER data, and reflecting the time period of this study, neither were tumor PDL-1 status and the use of immunotherapy. Finally, because this study was not validated in multiple centers, we could confirm the findings in our future large-scale multicenter prospective study.

In conclusion, unresectable patients with stage IIIA-N2 LCNEC and LUSC had worse LCSS compared with LUAD. In our study, the prognostic nomogram constructed for patients with unresectable NSCLC in stage IIIA-N2 could accurately predict survival by histological type, which may be a practical tool for clinicians to assess prognosis and stratify these prognostic risks, thus providing patients with more optimized and personalized treatment strategies based on histology.

## Author Contributions

YY and CS: data collection, data analysis, and drafting articles. JS: data collection. YW and GW: research conception, design, and interpretation. AS: manuscript revision and submit manuscript. All authors contributed to the article and approved the submitted version.

## Funding

The study was funded by the Scientific Research Project of “333 Project” in Jiangsu Province (BRA2019030), the Nantong Science and Technology Foundation (MS22019008), and the Nantong Municipal Health Commission scientific research project (QA2021028).

## Conflict of Interest

The authors declare that the research was conducted in the absence of any commercial or financial relationships that could be construed as a potential conflict of interest.

## Publisher’s Note

All claims expressed in this article are solely those of the authors and do not necessarily represent those of their affiliated organizations, or those of the publisher, the editors and the reviewers. Any product that may be evaluated in this article, or claim that may be made by its manufacturer, is not guaranteed or endorsed by the publisher.
