# Supplementary figures and images for: Based on the Development and Verification of a Risk Stratification Nomogram: Predicting the Risk of Lung Cancer-Specific Mortality in Stage IIIA-N2 Unresectable Large Cell Lung Neuroendocrine Cancer Compared With Lung Squamous Cell Cancer and Lung Adenocarcinoma
Source: Front Oncol. 2022 Jun 30;12:825598. doi: 10.3389/fonc.2022.825598 (PMC9282874; doi:10.3389/fonc.2022.825598)

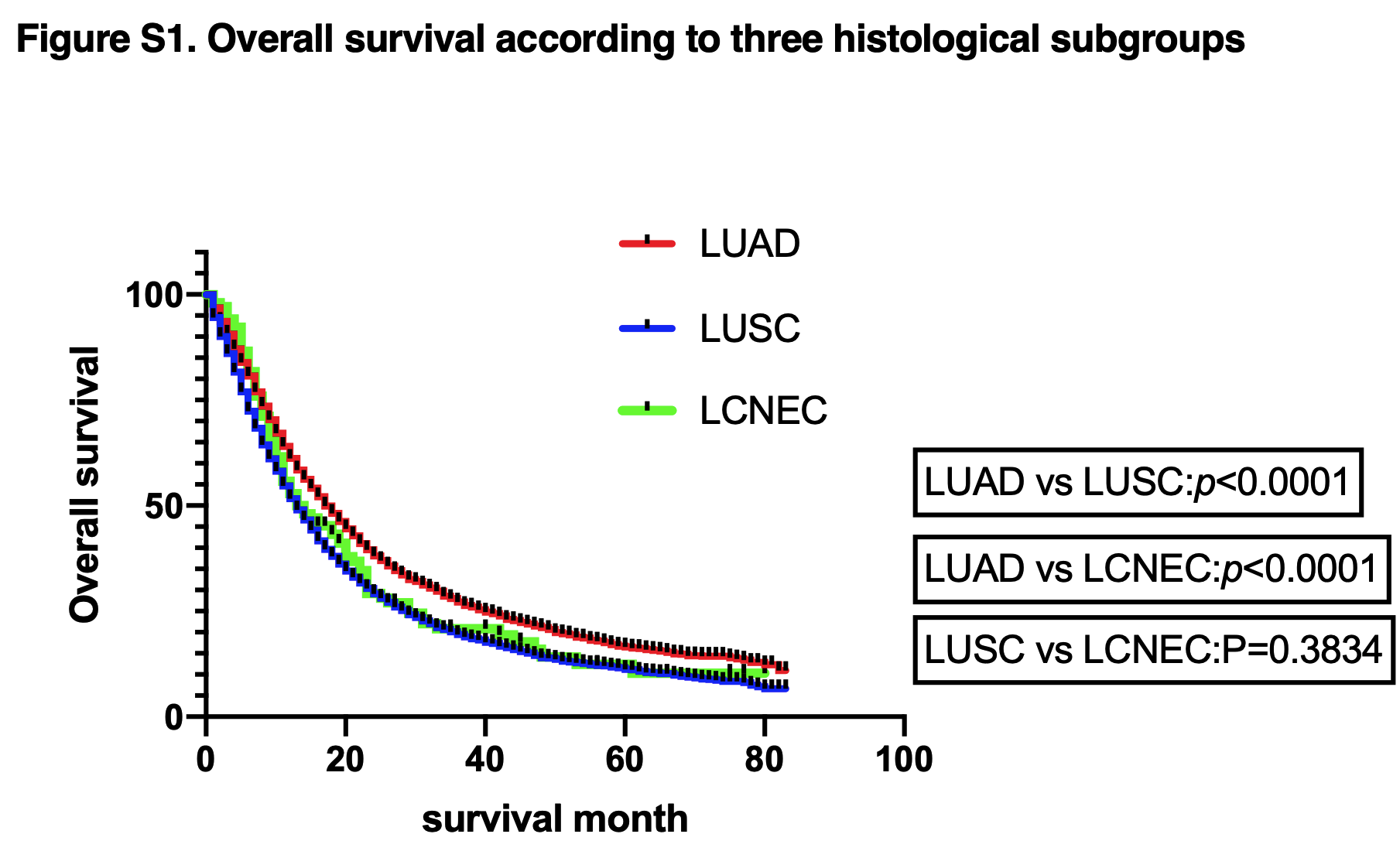

Supplement: Supplementary file 1 [file Image_1.tiff]

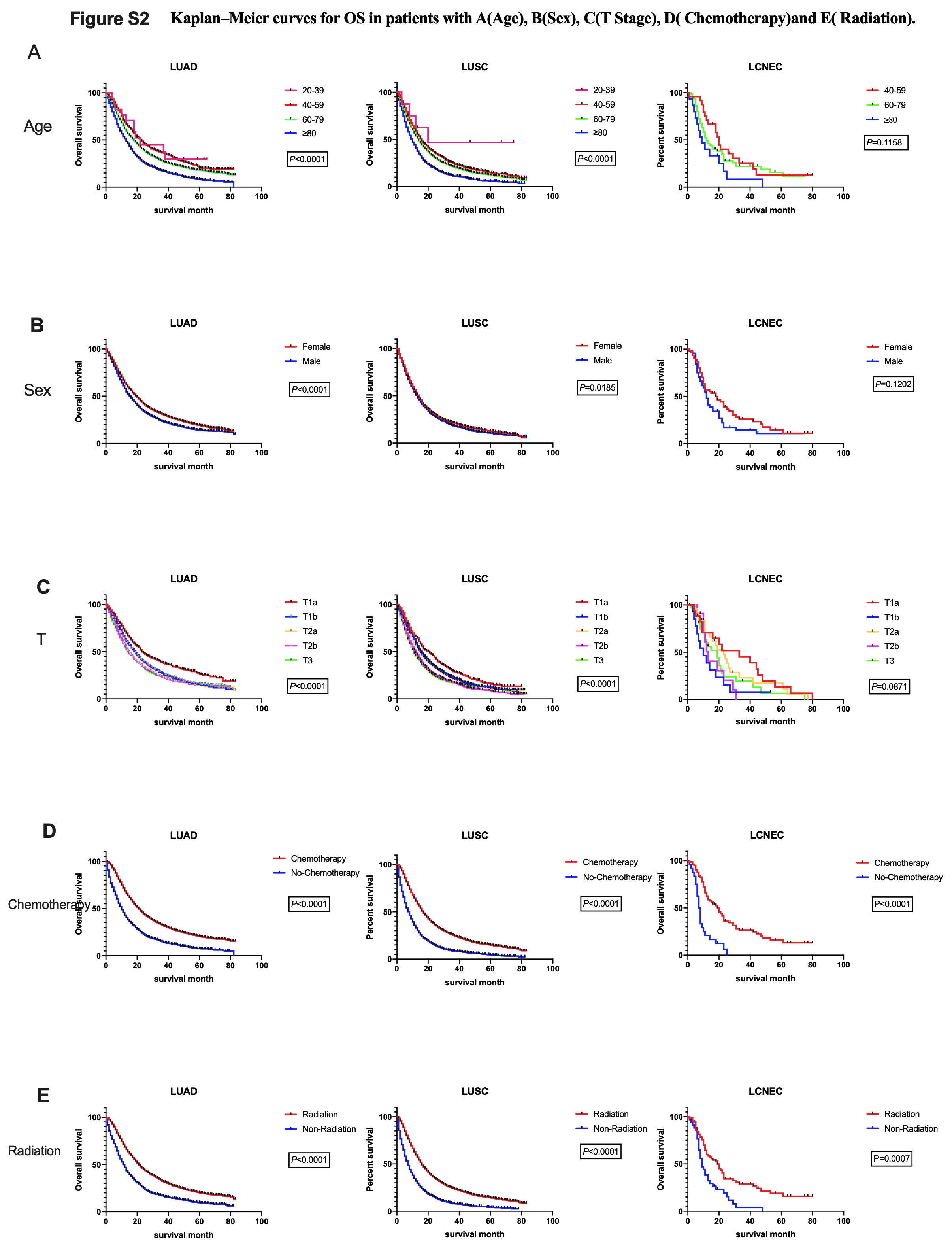

Supplement: Supplementary file 2 [file Image_2.tiff]
