# Supplementary material for: Based on the Development and Verification of a Risk Stratification Nomogram: Predicting the Risk of Lung Cancer-Specific Mortality in Stage IIIA-N2 Unresectable Large Cell Lung Neuroendocrine Cancer Compared With Lung Squamous Cell Cancer and Lung Adenocarcinoma
Source: Front Oncol. 2022 Jun 30;12:825598. doi: 10.3389/fonc.2022.825598 (PMC9282874; doi:10.3389/fonc.2022.825598)
Supplement: Supplementary file 3 [file Table_1.docx]

Table S1. Patients’ baseline characteristics according to three histological subgroups

| **Clinical Parameters** | | **LUAD**  **(n=5559)**  **N (%)** | **LUSC**  **(n=5842)**  **N (%)** | **LCNEC**  **(n=104)**  **N (%)** |  | ***P*** |  |  |
| --- | --- | --- | --- | --- | --- | --- | --- | --- |
| **Age** | 20-39 | 17 (0.3) | 8 (0.1) | / | <0.001 | | | |
|  | 40-59 | 1032 (18.6) | 832 (14.2) | 24（23.1） |  |  |  |  |
|  | 60-79 | 3441 (61.9) | 3838 (65.7) | 65（62.5） |  |  |  |  |
|  | ≥ 80 | 1069 (19.2) | 1164 (19.9) | 15（14.4） |  |  |  |  |
| **Sex** | Male | 2711 (48.8) | 3642 (62.3) | 44（42.3） | <0.001 | | | |
|  | Female | 2848 (51.2) | 2200 (37.7) | 60（57.7） |  |  |  |  |
| **Race** | Black | 798 (14.4) | 743 (12.7) | 17（16.3） | <0.001 | | | |
|  | White | 4363 (78.4) | 4802 (82.2) | 86（82.7） |  |  |  |  |
|  | Other | 398 (7.2) | 297 (5.1) | 1（1.0） |  |  |  |  |
| **Region** | East | 2509 (45.1) | 2958 (50.6) | 57（54.8） | <0.001 | | | |
|  | Northern Plains | 636 (11.4) | 640 (11.0) | 5（4.8） |  |  |  |  |
|  | Southern | 135 (2.4) | 147 (2.5) | 1（1.0） |  |  |  |  |
|  | Alaska | 4 (0.1) | 14 (0.2) | / |  |  |  |  |
|  | Pacific Coast | 2275 (40.9) | 2083 (35.7) | 41（39.4） |  |  |  |  |
| **Grade** | I | 253 (4.6) | 103 (1.8) | 1（1.0） | <0.001 | | | |
|  | II | 796 (14.3) | 1352 (23.1) | / |  |  |  |  |
|  | III | 1541 (27.7) | 1821 (31.2) | 33（31.7） |  |  |  |  |
|  | IV | 35 (0.6) | 33 (0.6) | 9（8.7） |  |  |  |  |
|  | Unknown | 2934 (52.8) | 2533 (43.4) | 61（58.7） |  |  |  |  |
| **Tumor Location** | Main bronchus | 137 (2.5) | 332 (5.7) | 4（3.8） | <0.001 | | | |
|  | Upper lobe | 3443 (61.9) | 3426 (58.6) | 63（60.6） |  |  |  |  |
|  | Middle lobe | 259 (4.7) | 221 (3.8) | 7（6.7） |  |  |  |  |
|  | Lower lobe | 1531 (27.5) | 1670 (28.6) | 25（24.0） |  |  |  |  |
|  | Overlapping | 21 (0.4) | 52 (0.9) | 1（1.0） |  |  |  |  |
|  | NOS | 168 (3.0) | 141 (2.4) | 4（3.8） |  |  |  |  |
| **T stage** | T1a | 791 (14.2) | 381 (6.5) | 17（16.3） | <0.001 | | | |
|  | T1b | 877 (15.8) | 490 (8.4) | 14（13.5） |  |  |  |  |
|  | T2a | 1577 (28.2) | 1613 (27.6) | 29（27.9） |  |  |  |  |
|  | T2b | 748 (13.5) | 1002 (17.2) | 11（10.6） |  |  |  |  |
|  | T3 | 1566 (28.2) | 2356 (40.3) | 33（31.7） |  |  |  |  |
| **Chemotherapy** | No/Unknown | 1644 (29.6) | 1986 (34.0) | 24（23.1） | <0.001 | | | |
|  | Yes | 3915 (70.4) | 3856 (66.0) | 80（76.9） |  |  |  |  |
| **Radiotherapy** | No/Unknown | 1885 (33.9) | 1694 (29.0) | 34（32.7） | <0.001 | | | |
|  | Yes | 3674 (66.1) | 4148 (71.0) | 70（67.3） |  |  |  |  |

LUAD: lung adenocarcinoma, LUSC: lung squamous cancer, LCNEC: large cell neuroendocrine cancer; *p*<0.05 is statistically significant.
